# Review of treehopper genus *Elaphiceps* Buckton, 1903 (Hemiptera, Membracidae, Centrotinae), with the first description of the male of *E. brachyspinus* Yuan & Chou, 1988

**DOI:** 10.3897/zookeys.1284.181440

**Published:** 2026-07-03

**Authors:** Feng-E Li, Jian-Kun Long, Zhi-Min Chang, Lin Yang, Xiang-Sheng Chen

**Affiliations:** 1 The Provincial Key Laboratory of Agricultural Biosecurity of Guizhou, Guizhou University, Guiyang, Guizhou, 550025, China Institute of Entomology, Guizhou University Guiyang China; 2 Institute of Entomology, Guizhou University, Guiyang, Guizhou, 550025, China The Provincial Key Laboratory of Agricultural Biosecurity of Guizhou, Guizhou University Guiyang China; 3 The Provincial Special Key Laboratory for Development and Utilization of Insect Resources of Guizhou, Guizhou University, Guiyang, Guizhou, 550025, China The Provincial Special Key Laboratory for Development and Utilization of Insect Resources of Guizhou, Guizhou University Guiyang China

**Keywords:** Complete record, diagnostic characteristic, distribution, scutellum, treehopper

## Abstract

A review of the genus *Elaphiceps* Buckton, 1903 (Hemiptera, Membracidae, Centrotinae) is presented. The genus comprises four species—*E. brachyspinus*, *E. cervus*, *E. neocervus*, and *E. javanensis*—a diagnosis, illustrations of the habitus, and the geographic distribution is provided for each. A diagnostic key is provided, and the male of *E. brachyspinus* is described for the first time.

## Introduction

The treehopper subfamily Centrotinae (Hemiptera, Membracidae) exhibits high diversity in China, with more than 280 species having been described (Yuan and Chou 2002; Cai and Xu 2004; Zeng 2005; Li et al. 2019, 2022). However, the tribal affiliation of several genera, including the genus *Elaphiceps* Buckton, 1903, cannot be confidently determined based on available morphological characters (e.g. pronotum and wing venation), leaving them designated as *incertae sedis* (Dmitriev et al. 2022). *Elaphiceps* was established by Buckton (1903) for the type species, *E. cervus* Buckton, 1903, from China. The genus later expanded to include *E. javanensis* Funkhouser, 1937 from Java, and two additional Chinese species described by Yuan and Chou (1988): *E. neocervus* and *E. brachyspinus*. Despite having morphological features that are distinct from other genera of Centrotinae, the tribal classification of *Elaphiceps* remains unresolved, and its tribal placement is dependent on a more comprehensive study of its morphological characters, particularly of the genitalia.

**Figure 1.**
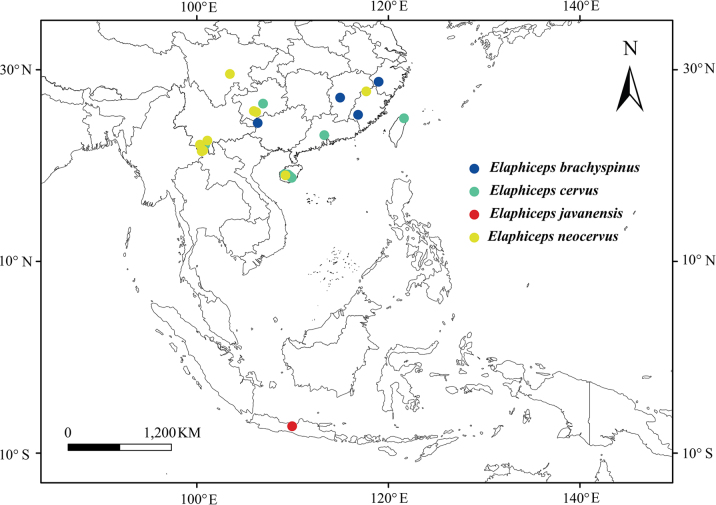
Geographic distributions of species of *Elaphiceps* Buckton, 1903: *E. brachyspinus* (GUGC: EB001–2; Yuan and Chou 2002; Dmitriev et al. 2022); *E. cervus* (GUGC: EC001–5; Yuan and Chou 2002; Dmitriev et al. 2022); *E. javanensis* (Dmitriev et al. 2022); *E. necervus* (GUGC: EN001–4; Yuan and Chou 2002; Dmitriev et al. 2022).

In this study, the diagnosis of *Elaphiceps* has been redefined. A key to species based on morphology is provided, as well as a map showing the geographic distributions of the species (Fig. 1). Four *Elaphiceps* species are described in detail, and the male of *E. brachyspinus* is described for the first time.

## Materials and methods

Morphological and biogeographical terminology follows Deitz (1975), Dietrich et al. (2001), Yuan and Chou (2002), Mejdalani (1998; female genitalia), and Holt et al. (2013). Habitus photographs were taken with a Canon EOS5D/MP-E 65 mm setup and focus-stacked using Helicon Focus v. 6. Wing images were photographed with a Keyence VHX-1000/6000 system and similarly processed. The genital segments of the specimens examined were cleared in 10% NaOH and drawn from preparations in glycerine jelly using a Leica MZ 12.5 stereomicroscope. The photographs and the illustrations were imported into Adobe Photoshop CS5 for composition and labelling of the figures. Examined specimens are deposited in the following institutions: Institute of Entomology, Guizhou University, Guiyang, China (**GUGC**), Department of Entomology, Smithsonian National Museum of Natural History, Washington, D.C., USA (**USNM**).

## Taxonomy

### Elaphiceps

Taxon classificationAnimaliaHemipteraMembracidae

Buckton, 1903

AD9BE895-C555-5E9F-B1BC-04A0FA15B1F0

Elaphiceps Buckton, 1903: 217—Yuan and Chou 1988: 72; Metcalf and Wade 1965: 106; Yuan and Chou 2002: 112; Wallace and Deitz 2004: 35.

#### Type species.

*Elaphiceps cervus* Buckton, 1903.

#### Diagnosis.

**Figure 2.**
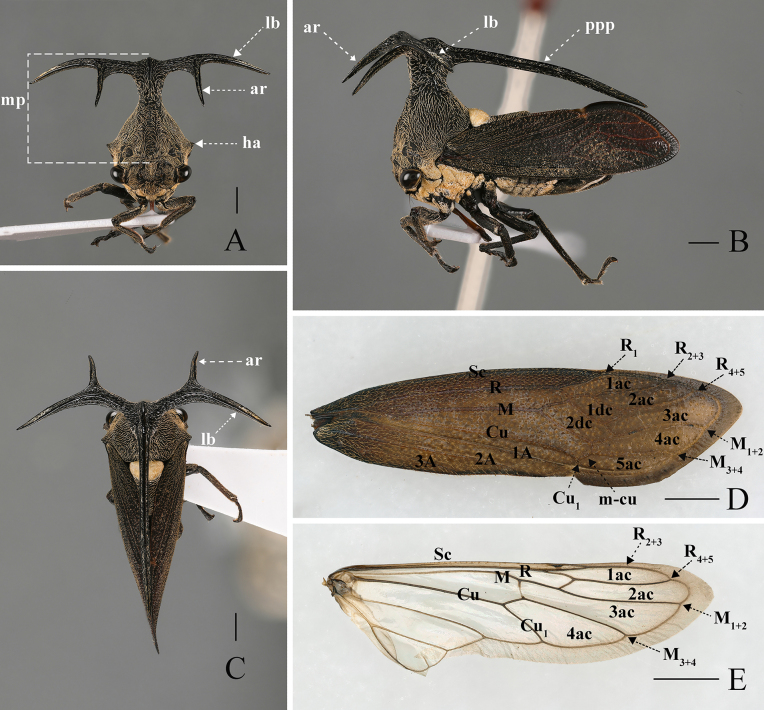
*Elaphiceps cervus* Buckton, 1903. **A**. Head and pronotum, anterior view; **B**. Habitus, lateral view; **C**. Habitus, dorsal view; **D**. Forewing; **E**. Hindwing. Scale bars: 0.5 mm. mp: mesodorsal process; lb: lateral branch; ar: anterior rebranch; ha: humeral angle; ppp: posterior pronotal process; ac: apical cells; dc: discoidal cells.

Medium-sized to large. Colouration black to brown. Body hairy. Head subquadrate; vertex with dorsal margins distinctly arcuate and ventral margins wavy or slightly oblique. Ocelli ovoid to globular, closer to inner margins of eyes than to each other. Frontoclypeal margins and lobes generally rhombic to ligulate; frontoclypeal lobes distinct. Pronotum black or reddish brown, coarsely punctate, sparsely pubescent. Humeral angles triangular, robust, with blunt or acute apices. Metopidium convex in lateral view; middle carina distinct. Mesodorsal process with base inversely funnel-shaped (Fig. 2A), apical portion columnar. In dorsal view, apex of mesodorsal process laterally extending to form lateral branches, giving entire structure a slightly curved outline (Fig. 2C). Lateral branches bear a pair of elongate, conical or triangular, anterior rebranches near their midlength (Figs 2C, 8C). Posterior pronotal process long, slender, triquerate, arising from summit of pronotum and extending beyond internal angles of forewing; profile slightly convex (Figs 2B, 5B, 9B) to concave (Fig. 8B). Scutellum entirely exposed, slender, with basal protuberance present; length longer than wide. Base with a semicircular patch of white to yellow pubescence; posterior margin emarginated with shallow arc, accounting for an extremely small proportion (Fig. 11B). Forewing nearly rectangular, dark brown to reddish brown, with pubescent; veins and wings same color. Basal quarter near Sc and R vein with opaque sclerotization; veins M+Cu and R fused basally, R and M fused with R_1_, R_2+3_ and M_1+2_ and M_3+4_, respectively; forewing with three crossveins present (one m-cu, one r-m, and one s), forming five apical cells and two discoidal cells (Fig. 2D). Hindwing with R vein branched into R_1_, R_2+3_, and R_4+5_, with vein M branched into M_1+2_ and M_3+4_, R_4+5_, and with M_1+2_ veins not fused, one r-m and one m-cu crossveins present (four apical cells present); apical limbus broad, with wrinkles (Fig. 2E). Metathoracic trochanter without spines; tibia with three rows of cucullate setae.

**Figure 3.**
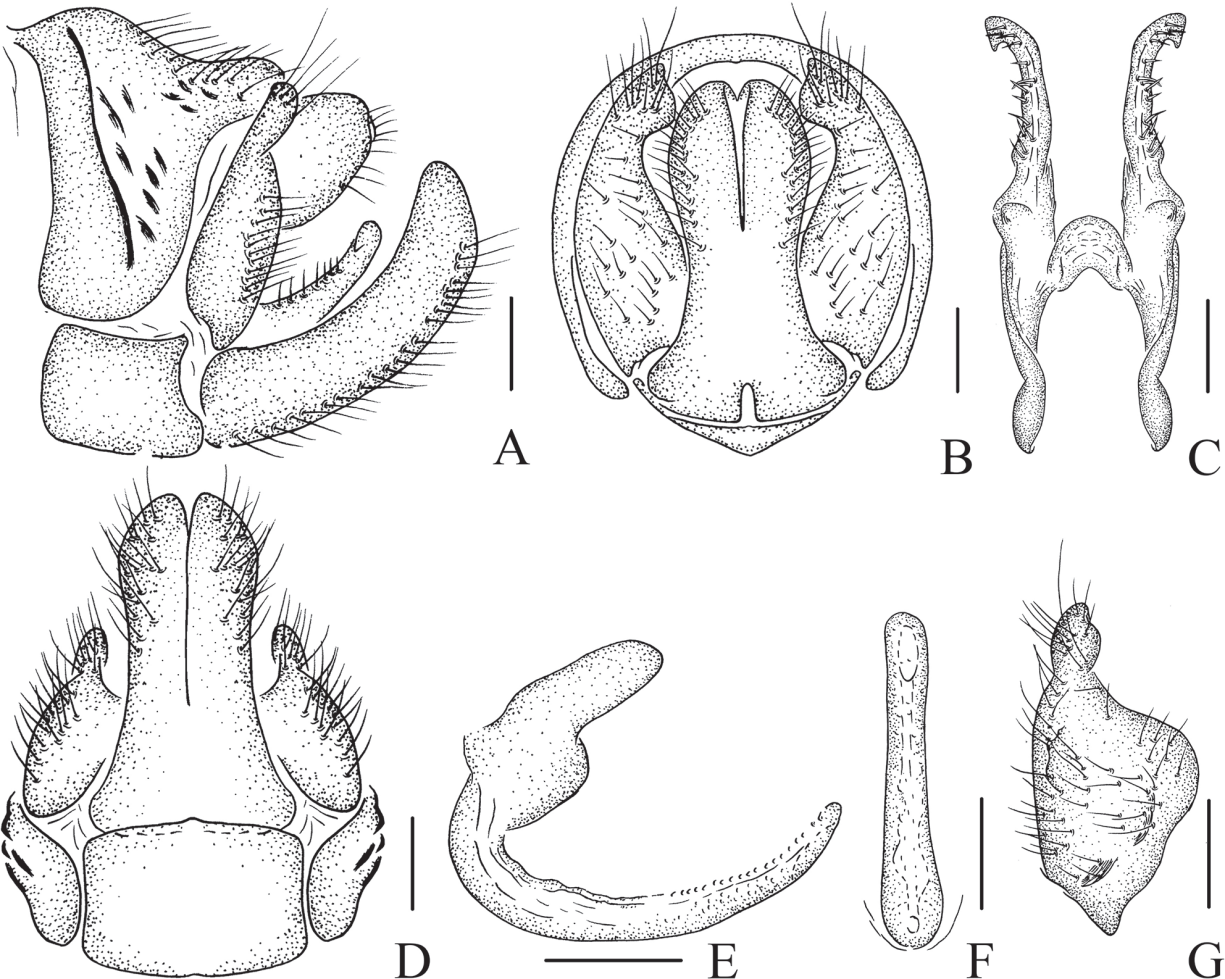
*Elaphiceps cervus* Buckton, 1903. **A, B, D**. Male genitalia, lateral, posterior, and ventral view, respectively; **C**. Style, dorsal view; **E, F**. Aedeagus, lateral, posterior view; **G**. Lateral plate. Scale bars: 0.2 mm.

***Male genitalia***. Pygofer nearly trapezoidal in lateral view, with dorsal margin convex basally, and apex slightly elongated, tubular (Figs 3A, 6A). Sternite IX rectangular, its ventral margin concave medially (Figs 3D, 6D). Lateral plate with a dorsoapical lobe dorsally extending; remainder triangular, setose (Figs 3G, 6G). Subgenital plates fused in basal half or slightly beyond (Figs 3D, 6D), slightly curled inward in the lateral view (Figs 3A, 6A). Style clasp laterally oriented (Figs 3A, 6A), triangular (Figs 3C, 6C); connective n-shaped, slightly expanded laterally. Aedeagus in lateral view nearly C-shaped; distal half with surface and margin bearing reverse serrations (Figs 3E, 6E); basal section tubular, formed by convergence toward centre of lamellar structures, with a wavy slit in middle (Figs 3E, 3F, 6E, 6F). In posterior view, gonopore apical, elongate-oval, occupying nearly one-quarter of total aedeagus length (Figs 3E, 3F, 6E, 6F).

**Figure 4.**
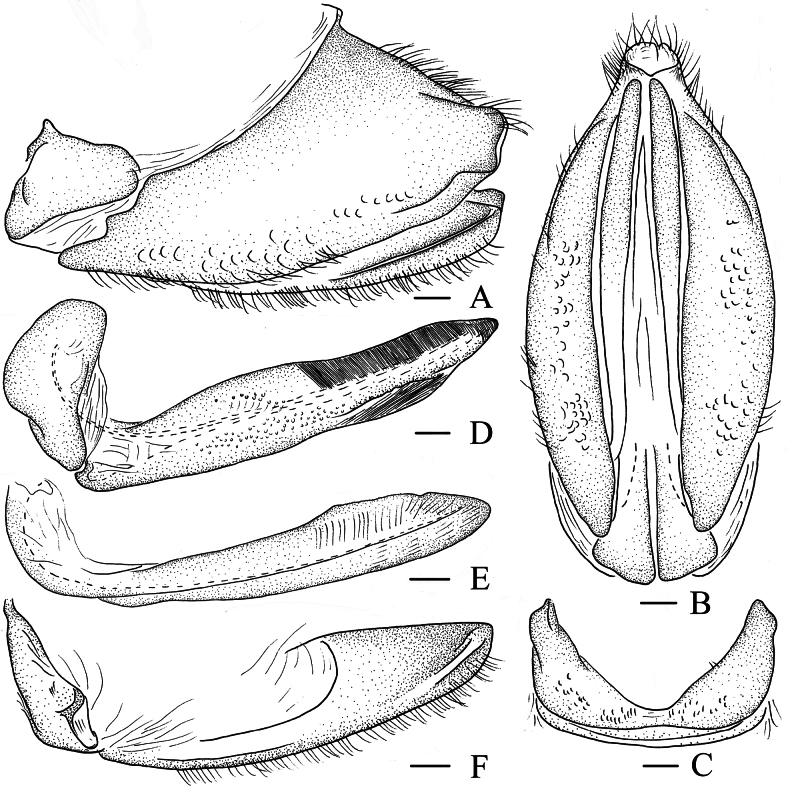
*Elaphiceps cervus* Buckton, 1903. **A**. Female genitalia, lateral view; **B**. Female genitalia, ventral view; **C**. Sternite VII, ventral view; **D**. Valvifer I and valvulae I, lateral view; **E**. Valvulae II, lateral view; **F**. Valvifer II and gonoplac, lateral view. Scale bar: 0.2 mm.

***Female genitalia***. Pygofer irregularly shoe-shaped in lateral view (base acuminate, apex tubular; Figs 4A, 7A, 10A) and elongate-oval in ventral view (Figs 4B, 7B, 10B). Sternite VII in ventral view with posterior margin deeply concave, bearing a triangular membranous area on each side (Figs 4C, 7C, 10C). Valvifer I semicircular, thin (Figs 4D, 7D, 10D); valvulae I knife-shaped, with sculptured lines on half or near half of dorsal margin surface and apical ventral margin (Figs 4D, 7D, 10D). Valvifer II shoe-shaped in lateral view (Figs 4F, 7F, 10F). Valvulae II knife-shaped, with apical third to half slightly widened; dorsal margin slightly convex or obliquely straight basally, serrated along basal half of widened portion and smooth distally (Figs 4E, 7E, 10E). Gonoplac with apical half expanded; ventral margin with setae (Figs 4F, 7F, 10F).

#### Distribution.

China, Indonesia.

##### Checklist and distributions of species of *Elaphiceps* Buckton, 1903

*E. brachyspinus* Yuan & Chou, 1988; China (Fujian, Guangxi, Jiangxi, Hainan, Zhejiang).

*E. cervus* Buckton, 1903; China (Guangdong, Hainan, Taiwan, Yunnan, Guizhou).

*E. javanensis* Funkhouser, 1937; Indonesia (Java).

*E. neocervus* Yuan & Chou, 1988; China (Hainan, Yunnan, Fujian, Sichuan, Guizhou).

##### Key to species of *Elaphiceps* Buckton, 1903

**Table:** 

1	Mesodorsal process with upper part short; metopidium obviously convex in lateral view; posterior pronotal process concave in lateral view (Fig. 8A–C)	***E. javanensis* Funkhouser, 1937**
–	Mesodorsal process with upper part long; metopidium slightly convex in lateral view; posterior pronotal process sloping and straight or slightly convex	**2**
2	Anterior rebranch obviously longer and straight, with apex slightly laterally extending, forming a π shape in head and pronotum in anterior view (Fig. 2A–C)	***E. cervus* Buckton, 1903**
–	Anterior rebranch short or only a slightly protruding and triangular	**3**
3	Anterior rebranch short and straight, slightly extending laterally (Fig. 9A–C)	***E. neocervus* Yuan & Chou, 1988**
–	Anterior rebranch extremely short and triangular (Fig. 5C)	***E. brachyspinus* Yuan & Chou, 1988**

### Elaphiceps cervus

Taxon classificationAnimaliaHemipteraMembracidae

Buckton, 1903

337310D1-FA56-53C7-9DBD-50C7DE7F5365

Figs 2, 3, 4

Elaphiceps cervus Buckton, 1903: 217—Funkhouser 1929: 478; Yuan 1986: 154; Yuan and Chou 1988: 75; Yuan and Chou 2002: 113–114; Wallace and Deitz 2004: 35.

#### Material examined.

• 1♀ (GUGC), China, Guizhou Province, Qiannan Buyi and Miao Autonomous Prefecture, Longli Country, Longjia Mountain Forest Park, 22-VII-2017, Xiang-Sheng Chen leg (no. EC001); • 1♂ (GUGC), China, Hainan Province, Baisha Li Autonomous County, Yinggeling National Nature Reserve, 6-IV-2017, Ying-Jian Wang leg (no. EC002); • 1♂ (GUGC), China, Hainan Province, Lingshui Li Autonomous County, Diaoluoshan National Nature Reserve, 15-IV-2017, Hong-Xing Li leg (no. EC003); • 1♀ (GUGC), China, Hainan Province, Changjiang Li Autonomous County, Bawangling National Nature Reserve, 2-V-2017, Hong-Xing Li leg (no. EC004); • 1♀ (GUGC), China, Hainan Province, Wuzhishan City, Wuzhishan National Nature Reserve, 17-IV-2017, Bin Yan leg (no. EC005).

#### Description.

Body length: male 9.2–9.5 mm (*n* = 2), female 9.7–10.1 mm (*n* = 3); forewing length: male 8.0–8.1 mm (*n* = 2), female 8.0–8.5 mm (*n* = 3); width between humeral angles apices: male 3.2–3.4 mm (*n* = 2), female 3.4–3.5 mm (*n* = 3); the length of anterior rebranch: male 2.0–2.3 mm (*n* = 2), female 2.0–2.1 mm (*n* = 3).

***Colouration***. General colour black, covered with yellow setae (Fig. 2A–C). Eyes black-brown, with a paler brown marginal ring. Forewing black-brown. Abdomen black; sternites with white and yellow pubescence. Legs black.

***Head and thorax***. Frontoclypeal margins and lobes with an overall rhombus-shape; apical margin arcuate (Fig. 2A). Mesodorsal process tall and slender (Fig. 2A, B). In dorsal view, lateral branch convex near its midlength and curve; length approximately three times that of anterior rebranch. Dorsal surface with a distinct median carina and two weak ridges (Fig. 2C). In lateral view, anterior rebranch extends well beyond metopidium (Fig. 2C). Posterior pronotal process long, slender, and slightly inclined posteriorly, extending beyond apex of first apical cell, with apex nearly touching forewing margins (Fig. 2B).

***Male genitalia***. Subgenital plates fused in basal half. Aedeagus with distal half bearing reverse serrations on its surface and margin. (Fig. 3E, F).

***Female genitalia***. The valvulae II knife-shaped, with apical third slightly widened and dorsal margin obliquely straight basally (Fig. 4E).

#### Host.

*Castanta mollissinma*; *Castanopsis diversifolia* (Fagaceae).

#### Distribution.

China (Guangdong, Guizhou, Hainan, Taiwan, Yunnan).

#### Note.

A new distribution record is reported here from Guizhou Province.

### Elaphiceps brachyspinus

Taxon classificationAnimaliaHemipteraMembracidae

Yuan & Chou, 1988

C783C4F8-35DF-5D95-8AF9-E2F750600289

Figs 5, 6, 7

Elaphiceps brachyspinus Yuan & Chou, 1988: 75—Yuan 1993: 366; Yuan and Chou 2002: 115–116.

#### Material examined.

• 1♂ (GUGC), China, Hainan Province, Leduo Li Autonomous County, Jianfengling National Nature Reserve, 25-IV-2017, Liang-Jing Yang leg (no. EB001); • 1♀ (GUGC), China, Zhejiang Province, Quzhou City, Qujiang District, Medicine King Mountain, 28-V-2020, light trapping Xiao-Han Ye leg (no. EB002).

#### Description.

Body length: male 11.5 mm (*n* = 1), female 11.9 mm (*n* = 1); forewing length: male 9.0 mm (*n* = 1), female 9.4 mm (*n* = 1); width between humeral angles apices: male 3.6 mm (*n* = 1), female 3.7 mm (*n* = 1); the length of anterior rebranch: male 0.5 mm (*n* = 1), female 0.7 mm (*n* = 1).

**Figure 5.**
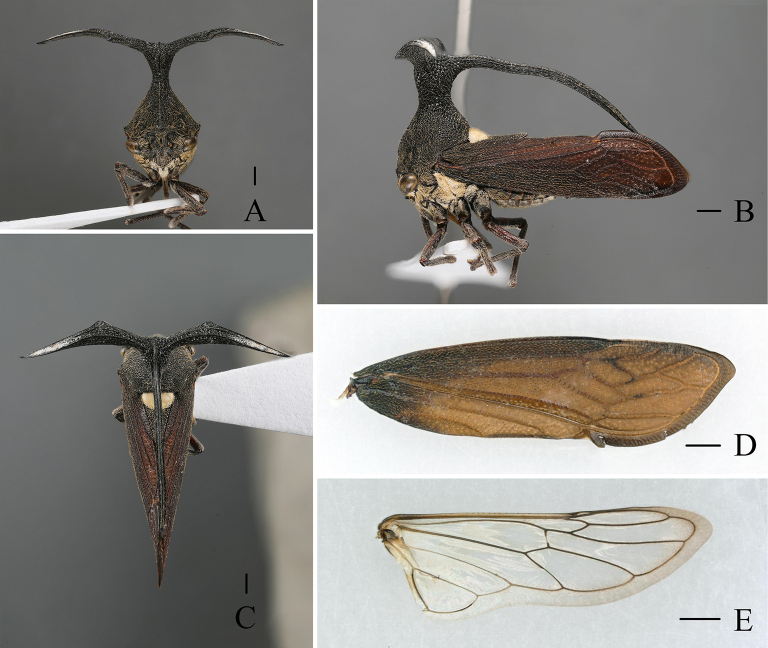
*Elaphiceps brachyspinus* Yuan & Chou, 1988. **A**. Head and pronotum, anterior view; **B**. Habitus, lateral view; **C**. Habitus, dorsal view; **D**. Forewing; **E**. Hindwing. Scale bars: 0.5 mm.

***Colouration***. General colour black, with yellow setae (Fig. 5A–C). Eyes dark yellow-brown. Forewing black, with brown areas. Abdomen black, with yellow pubescence; apex with yellowish brown stripes. Legs black except femur reddish brown.

***Head and thorax***. Vertex with dorsal margins bow-shaped; ventral margins undulate (Fig. 5A). Frontoclypeal margins apically arcuate (Fig. 5A). Mesodorsal process tall and slender (Fig. 5B). In dorsal view, lateral and anterior rebranches bow-shaped. Lateral branch with distinct carina and weak ridge; carinae overall parallel (Fig. 5C). Anterior rebranch triangular and short (Fig. 5C). Posterior pronotal process curved, extending nearly to apex of first apical cell; apex contacting forewing margins (Fig. 5B).

**Figure 6.**
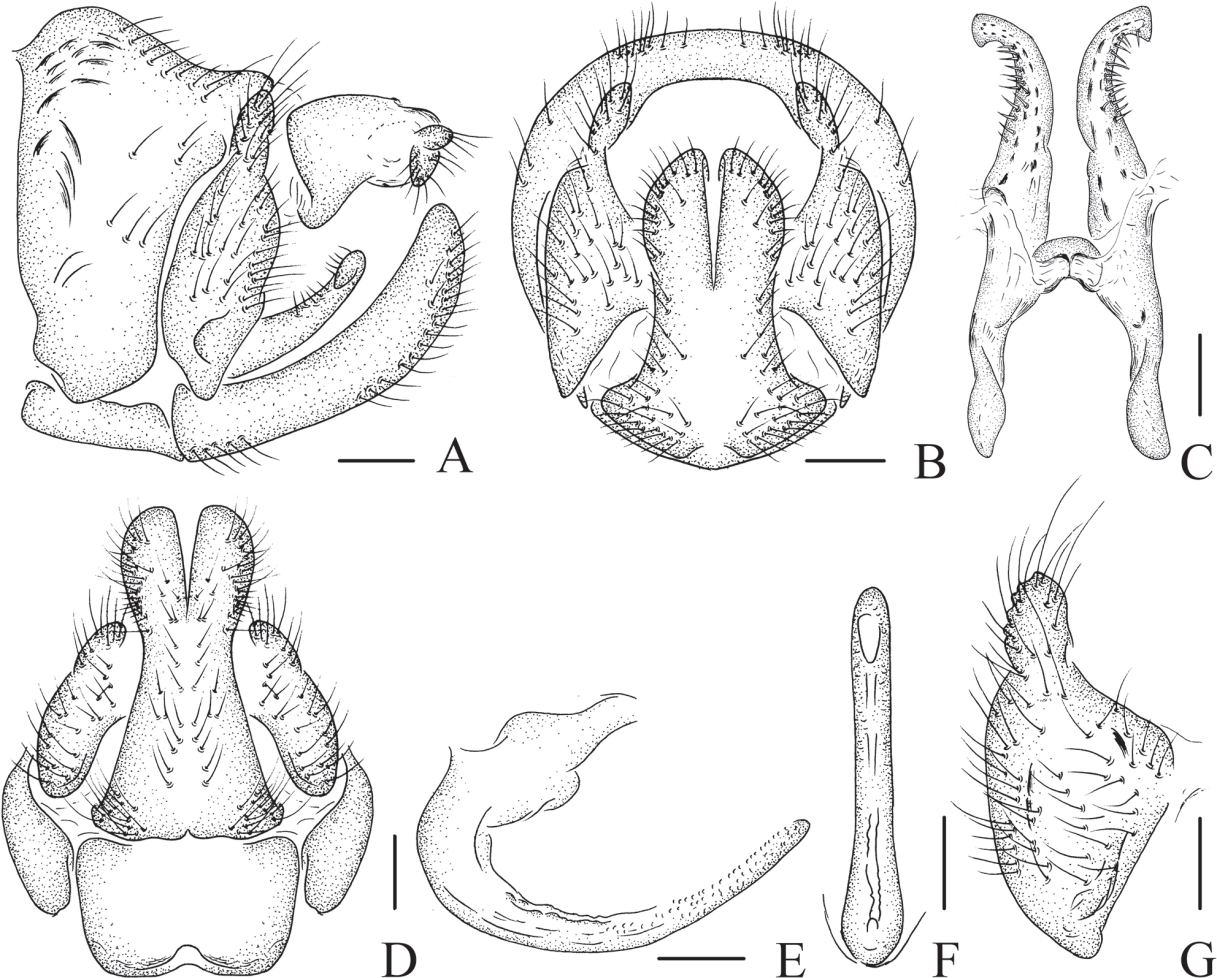
*Elaphiceps brachyspinus* Yuan & Chou, 1988. **A, B, D**. Male genitalia, lateral, posterior and ventral view; **C**. Style, dorsal view, **E, F**. Aedeagus, lateral, posterior view; **G**. Lateral plate. Scale bars: 0.2 mm.

***Male genitalia***. Subgenital plates basally fused for more than half their length (Fig. 6D). Aedeagus slightly medially swollen and narrow at both ends in lateral view (Fig. 6E, F).

**Figure 7.**
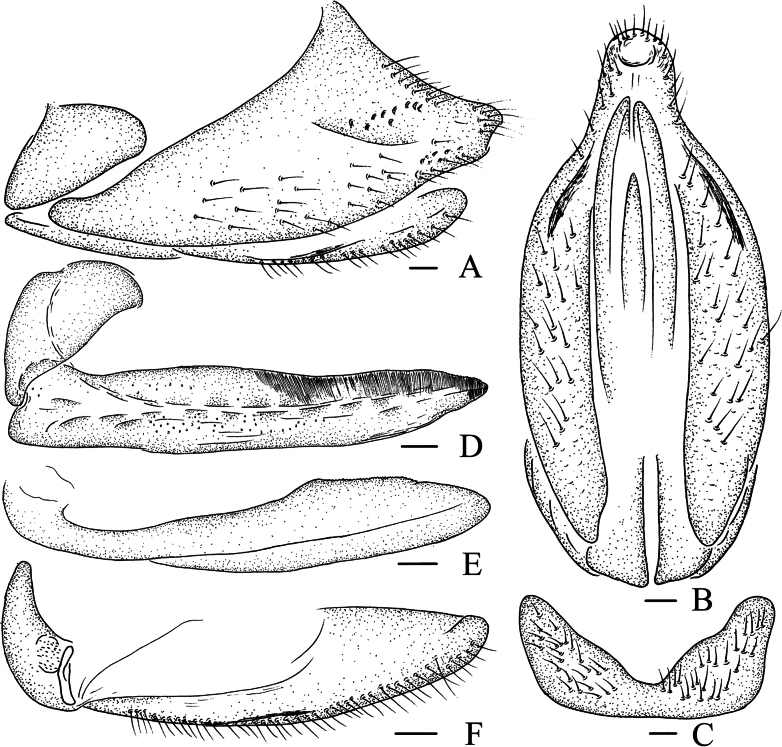
*Elaphiceps brachyspinus* Yuan & Chou, 1988. **A**. Female genitalia, lateral view; **B**. Female genitalia, ventral view; **C**. Sternite VII, ventral view; **D**. Valvifer I and valvulae I, lateral view; **E**. Valvulae II, lateral view; **F**. Valvifer II and gonoplac, lateral view. Scale bar: 0.2 mm.

***Female genitalia***. Valvulae II knife-shaped, apex submedially slightly widened; dorsal margin basally obliquely straight (Fig. 7E).

#### Distribution.

China (Fujian, Guangxi, Jiangxi, Hainan, Zhejiang).

#### Note.

Notably, the male is described here for the first time, and the species is newly recorded from Hainan and Zhejiang Provinces, which constitute new distribution records.

### Elaphiceps javanensis

Taxon classificationAnimaliaHemipteraMembracidae

Funkhouser, 1937

0D4322A6-8C41-52AF-B0A7-8A2B6AEEE892

Fig. 8

Elaphiceps javanensis Funkhouser, 1937: 121–122.

#### Description.

**Figure 8.**
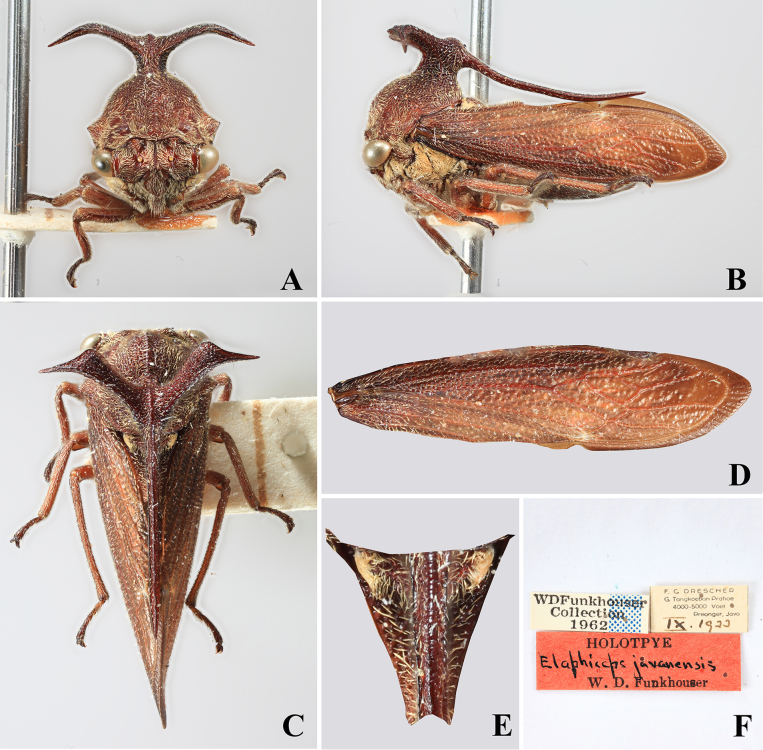
*Elaphiceps javanensis* Funkhouser, 1937. **A**. Head and pronotum, anterior view; **B**. Habitus, lateral view; **C**. Habitus, dorsal view; **D**. Forewing; **E**. Scutellum; **F**. Labels. Photos: Gary Ouellette, © Department of Entomology, Smithsonian National Museum of Natural History (USNM), http://n2t.net/ark:/65665/3965d9e0e-554e-4572-8dc8-a31898b4d4c6.

***Colouration***. General colour reddish brown, with gold hairs (Fig. 8A–C). Eyes pale yellow-brown; ocelli orange-yellow. Thorax brown, with yellow pubescence. Legs pale brown; tarsi and claws dark brown.

***Head and thorax***. Head with longitudinal median carina; vertex with dorsal margins distinctly bow-shaped; ventral margins undulate (Fig. 8A). Frontoclypeal margins apically triangular (Fig. 8A). Metopidium strongly convex in lateral view (Fig. 8B). Mesodorsal process short and stocky (Fig. 8A). In dorsal view, lateral branch extends laterally and dorsally, then runs slightly downward. Anterior rebranch triangular, with curved apex (Fig. 8C). Posterior pronotal process arced and inwardly concave; apex extending beyond midpoint of first apical cell and contacting forewing margins along approximately one-third of its length (Fig. 8B).

#### Distribution.

Indonesia (Java).

#### Note.

The present interpretation of the species is based on photographs of the holotype provided by the Department of Entomology, Smithsonian National Museum of Natural History (USNM).

### Elaphiceps neocervus

Taxon classificationAnimaliaHemipteraMembracidae

Yuan & Chou, 1988

88F98E37-BFC4-53C4-86F6-1C9ED02D7E6A

Figs 9, 10

Elaphiceps necervus Yuan & Chou, 1988: 75—Yuan 1993: 366; Yuan and Chou 2002: 114–115.

#### Material examined.

• 1♀ (GUGC), China, Guizhou Province, Anshun City, Ziyun Miao and Bouyei Autonomous County, Nazuo Village, 8-VI-2019, Yong-Qing Fang leg (no. EN001); • 1♀ (GUGC), China, Guizhou Province, Anshun City, Ziyun Miao and Bouyei Autonomous County, Luga Village, 3-VII-2019, Yong-Qing Fang leg (no. EN002); • 1♀ (GUGC), China, Yunnan Province, Puer City, Simao District, Puer Sun River National Park, 27-VI-2019, Feng-E Li leg (no. EN003); • 1♀ (GUGC), China, Hainan Province, Leduo Li Autonomous County, Jianfeng Town, Hainan Jianfengling National Nature Reserve, 12-V-2021, Lan Zhang leg (no. EN004).

#### Description.

Body length: female 11.5–12.1 mm (*n* = 4); forewing length: female 10.5–10.6 mm (*n* = 4); width between humeral angles apices: female 4.0–4.1 mm (*n* = 4); length of anterior rebranch: female 1.5–1.7 mm (*n* = 4).

**Figure 9.**
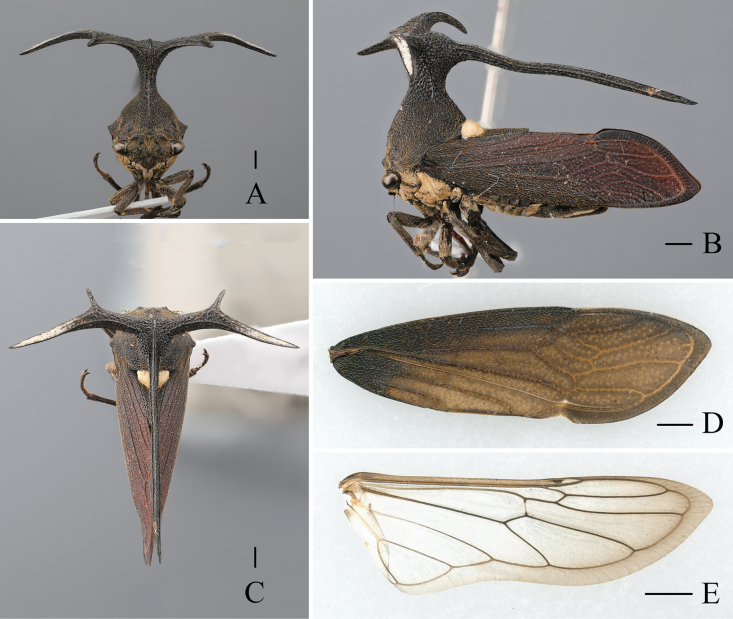
*Elaphiceps necervus* Yuan & Chou, 1988. **A**. Head and pronotum, anterior view; **B**. Habitus, lateral view; **C**. Habitus, dorsal view; **D**. Forewing; **E**. Hindwing. Scale bars: 0.5 mm.

***Colouration***. General colour black, with yellow setae (Fig. 9A–C). Eyes yellow-brown, with black maculae; ocelli dark orange. Forewing black, infused with reddish brown. Abdomen, with white and yellow pubescence. Legs black.

***Head and thorax***. Vertex with dorsal margins bow-shaped; ventral margins undulate. Frontoclypeal margins apically rather blunt (Fig. 9A). Mesodorsal process tall and slender (Fig. 9B). In dorsal view, lateral and anterior rebranches bow-shaped. Lateral branch with a distinct carina and a weak ridge. Anterior rebranch elongate, horn-like, about one-fifth of length of lateral branch (Fig. 9C). Posterior pronotal process slightly curved to almost straight, its apex slightly distant from forewing margins (Fig. 9B).

**Figure 10.**
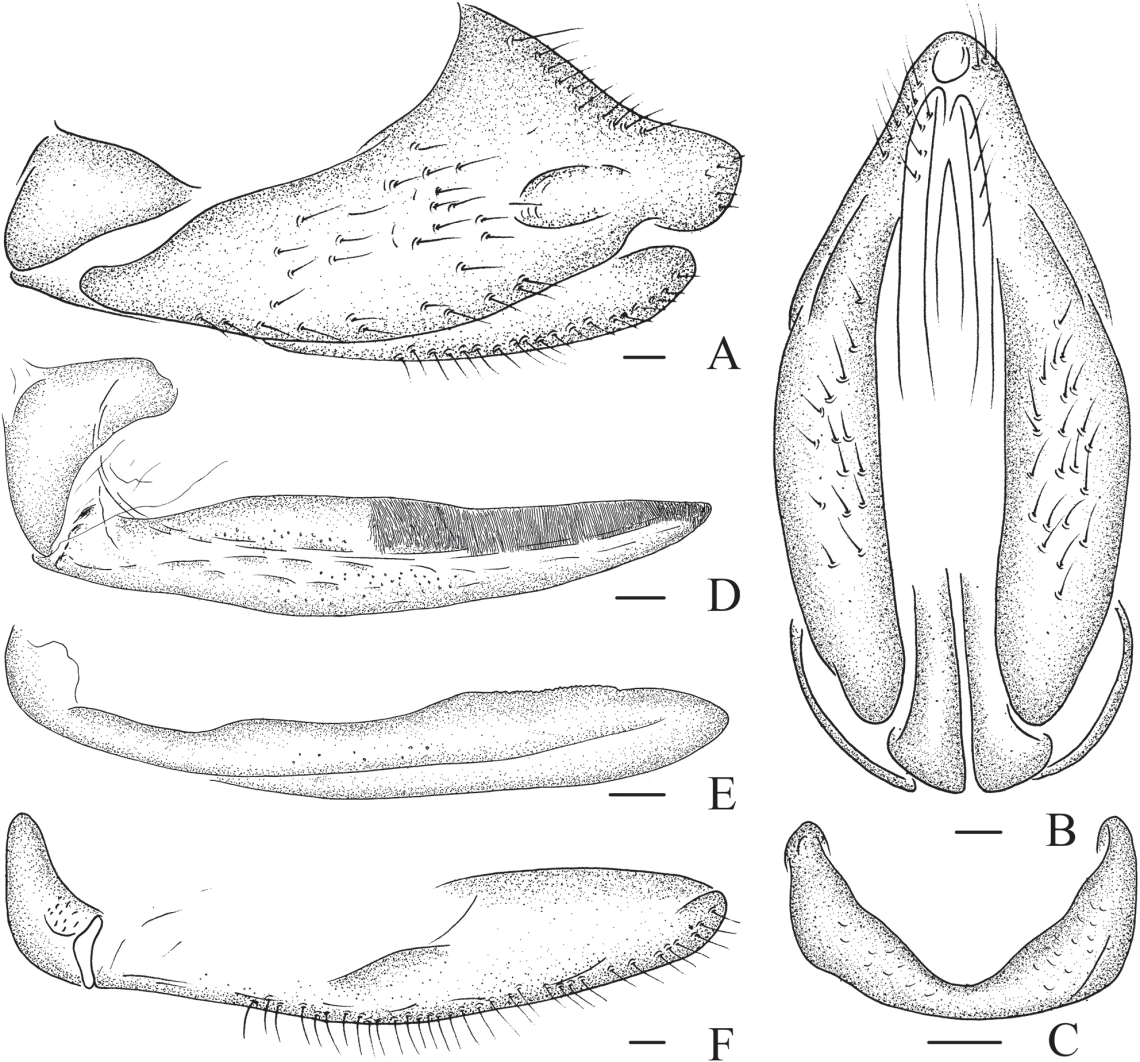
*Elaphiceps necervus* Yuan & Chou, 1988. **A**. Female genitalia, lateral view; **B**. Female genitalia, ventral view; **C**. Sternite VII, ventral view; **D**. Valvifer I and valvulae I, lateral view; **E**. Valvulae II, lateral view; **F**. Valvifer II and gonoplac, lateral view. Scale bar: 0.2 mm.

***Female genitalia***. Valvulae II knife-shaped, with apical half slightly widened; dorsal margin slightly convex at basal quarter (Fig. 10E).

#### Distribution.

China (Fujian, Guizhou, Hainan, Sichuan, Yunnan).

#### Note.

New distribution records are reported from Hainan and Guizhou Provinces.

## Discussion

All known species of the genus *Elaphiceps* are distributed exclusively within the Oriental Realm (Holt et al. 2013), with China hosting the highest diversity of three recorded species; another species is found in Indonesia. This genus occurs predominantly in the regions of South China. The discovery of these additional distribution areas (Guizhou, Hainan, Zhejiang) reveals an underestimation of the actual range for most species within this genus, necessitating further comprehensive research. Furthermore, understanding of the host range for this genus remains scarce. This is evident for *E. cervus*, which has only been recorded on two host species to date (the genera *Castanta* and *Castanopsis*), and adults were found to be sparsely distributed among the branches and leaves within the canopy of tall trees (Yuan and Chou 2002). Despite repeated collection from the canopy during field surveys, the host plants for this genus have not been definitively identified. Actually, the presence of this genus in the canopy is unsurprising. *Elaphiceps* species are large and have a narrow pronotum, which is elongated with numerous branches. This morphology requires a large ecological space and a broad range of habitat. The canopy is characterized by having an open structure and, therefore, forms an ecological niche distinct from soil and shrub habitats (Wang et al. 2022). Future efforts should focus on accurately identifying host plants and further exploring the ecological interactions of this genus within the canopy.

### The taxonomic status of the genus

**Figure 11.**
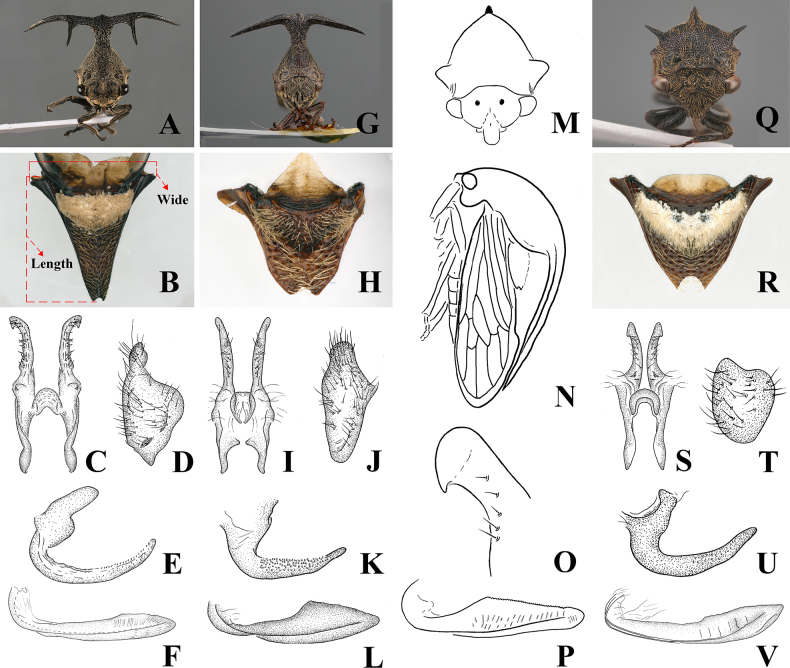
Comparative characteristics of genera *Elaphiceps*, *Leptobelus*, *Tyrannotus*, *Lobocentrus*. **A–F**. *Elaphiceps cervus*; **G–L**. *Leptobelus gazella*; **M–P**. *Tyrannotus tyrannicus* (based on Wallace and Deitz 2004, figs 0.11B, D, J, 0.12D); **Q–V**. *Lobocentrus triangularis*. **A, G, M, Q**. Head and pronotum, anterior view; **B, H, R**. Scutellum, dorsal view; **N**. Habitus, lateral view; **C, I, S, O**. Style; **D, J, T**. Lateral plate; **E, K, U**. Aedeagus; **F, L, P, V**. Valvulae II.

The current placement of the genus *Elaphiceps* is within the Centrotinae, where it is regarded as *incertae sedis* (Wallace and Deitz 2004; Dmitriev et al. 2022). The genus was placed in the Leptobelini by Yuan and Chou (2002). Although *Elaphiceps* shares similar pronotum features with *Leptobelus*, the male genitalia (lateral plate, aedeagus), female genitalia (valvulae II), and scutellum differ significantly. Additionally, morphological evidence supports that the genus *Tyrannotus* and tribe Lobocentrini forms a clade with the genus *Elaphiceps* (Wallace and Deitz 2004); however, these four genera have high morphological differentiation: for example, pronotum, scutellum, as well as the male genitalia with style, lateral plate, and aedeagus, and the female genitalia with valvulae II (Fig. 11). Despite the lack of molecular data, the genus may still be considered a candidate for elevation to the tribe level due to its significant uniqueness.

## Supplementary Material

XML Treatment for Elaphiceps

XML Treatment for Elaphiceps cervus

XML Treatment for Elaphiceps brachyspinus

XML Treatment for Elaphiceps javanensis

XML Treatment for Elaphiceps neocervus

## Additional information

### Artificial Intelligence (AI) use

The authors accept full responsibility for the content of the manuscript, including the disclosure of any use of AI.

No AI tools were used in the preparation of this manuscript.

### Author ORCIDs

F.-E. Li https://orcid.org/0000-0002-8529-3302

J.-K. Long https://orcid.org/0000-0002-4576-9919

Z.-M. Chang https://orcid.org/0000-0002-7247-9706

L. Yang https://orcid.org/0000-0002-7841-5156

X.-S. Chen https://orcid.org/0000-0001-9801-0343

### Data availability

All of the data that support the findings of this study are available in the main text.

## References

[B1] Buckton GB (1903) A Monograph of the Membracidae. L. Reeve & Co., Limited. London, 181–296. 10.5962/bhl.title.34753

[B2] Cai P, Xu RX (2004) A new species of the genus *Antialcidas* Distant (Hemiptera: Membracidae) from China. Entomotaxonomia 26(4): 261–263.

[B3] Deitz LL (1975) Classification of the higher categories of the New World treehopper (Homoptera: Membracidae). North Carolina Agricultural Experiment Station Technical Bulletin 225: 1–177.

[B4] Dietrich CH, McKamey SH, Deitz LL (2001) Morphology-based phylogeny of the treehopper family Membracidae (Hemiptera: Cicadomorpha: Membracoidea). Systematic Entomology 26: 213–239. 10.1046/j.1365-3113.2001.00140.x

[B5] Dmitriev DA, Angelova R, Anufriev GA, Bartlett CR, Blanco-Ro dríguez E, Borodin OI, Cao YH, Cara C, Deitz LL, Dietrich CH, Dmitrieva MO, El-Sonbati SA, Evangelista de Souza O, Gjonov IV, Gonçalves AC, Gonçalves CC, Hendrix SV, McKamey S, Kohler M, Kunz G, Malenovský I, Morris BO, Novoselova M, Pinedo-Escatel JA, Rakitov RA, Rothschild MJ, Sanborn AF, Takiya DM, Wallace MS, Zahniser JN (2022) *Elaphiceps* Buckton, 1903. World Auchenorrhyncha Database. TaxonPages. https://hoppers.speciesfile.org/otus/51462/overview [accessed on 14 November 2025]

[B6] Funkhouser WD (1929) The Membracidae of China. Lingnan Journal of Science 7: 475–479.

[B7] Funkhouser WD (1937) Fauna Javanensis. Membracidae (Homoptera). Tijdschrift Voor Entomologie 80: 121–126.

[B8] Holt BG, Lessard JP, Borregaard MK, Fritz SA, Araujo MB, Dimitrov D, Fabre PH, Graham CH, Graves GR, Jonsson KA, Nogues-Bravo D, Wang ZH, Whittaker RJ, Fjeldsa J, Rah bek C (2013) An update of Wallace’s zoogeographic regions of the world. Science 339(6115): 74–78. 10.1126/science.1228282 23258408

[B9] Li FE, Yang L, Long JK, Chang ZM, Chen XS (2019) A review of the genus *Sinocentrus* Yuan (Hemiptera, Membracidae, Centrotinae) with description of a new species from China. ZooKeys 886: 135–144. 10.3897/zookeys.886.36672 PMC6851225 31736626

[B10] Li FE, Yang L, Long JK, Chang ZM, Chen XS (2022) Two new species of the treehopper genus *Machaerotypus* Uhler, 1896 from China (Hemiptera: Membracidae: Centrotinae). European Journal of Taxonomy 826: 64–79. 10.5852/ejt.2022.826.1835

[B11] Mejdalani G (1998) Morfologia externa dos Cicadellinae (Homoptera, Cicadellidae): comparação entre *Versigonalia ruficauda* (Walker) (Cicadellini) e *Tretogonia cribrata* Meli char (Proconiini), com notas sobre outras espécies e análise da terminologia. Revista Brasileira de Zoologia 15: 451–544. 10.1590/S0101-81751998000200015

[B12] Metcalf ZP, Wade V (1965) General Catalogue of the Homoptera. A Supplement to Fascicle I – Membracidae of the General Catalogue of the Hemiptera. Membracoidea in Two Sections. Section I. Part 1 – Membracidae: Centrotinae, Platybelinae, Hoplophorioninae, Darninae. Waverly Press, Baltimore, 743 pp.

[B13] Wallace MS, Deitz LL (2004) Phylogeny and systematics of the treehopper subfamily Centrotinae (Hemiptera: Membracidae). Memoirs on Entomology International 19: 1–377.

[B14] Wang MQ, Luo AR, Zhou QS, Chen JT, Xie TT, Li Y, Chesters D, Shi XY, Xiao H, Liu HJ, Ding Q, Zhou X, Luo YP, Lu YY, Tong YJ, Zhao ZY, Bai M, Guo PF, Chen SC, Nakamura A, Peng YQ, Zhao YH, Wei SH, Lin XL, Chen HY, Luo SX, Liu YH, Liu L, Yu JP, Zhou X, Zou Y, Lu H, Zhu CD (2022) Research progress on insect diversity. Biodiversity Science 30: 22454. 10.17520/biods.2022454

[B15] Yuan F (1986) Agricultural Insects of China (Volume 1). Agricultural Press, Beijing.

[B16] Yuan F (1993) Rare insects of Wuyishan Nature Reserve: *Elaphiceps* and *Hypsauchenia* (Hemiptera: Membracidae). In: Zhao Fuxiu (Ed.) Scientific Survey Report of Wuyishan Nature Reserve. Fujian Science and Technology Press, Fuzhou, 366–368.

[B17] Yuan F, Chou I (1988) Advances in the research of systematics of Membracoidea from China (Homoptera, Auchenorrhyncha). In: Vidano C, Arzone A (Eds) 6^th^Auchenorrhyncha Meeting, Turin, Italy, September 7–11, 1987, Proceedings. Consiglio Nazionale delle Ricerche. Incremento Produttività Risorse Agricole (CNR-IPRA), Torino, 71–78.

[B18] Yuan F, Chou I (2002) Fauna Sinica. Insecta (Vol. 28). HomopteraMembracoidea. Science Press, Beijing, 590 pp.

[B19] Zeng Y (2005) A new species of the genus *Maurya* Distant (Hemiptera: Membracidae) from China. Entomotaxonomia 27(4): 266–268.

